# Assessment of geographical origin of virgin coconut oil using inductively coupled plasma mass spectrometry along with multivariate chemometrics

**DOI:** 10.1016/j.crfs.2022.03.003

**Published:** 2022-03-11

**Authors:** Amit, Rahul Jamwal, Shivani Kumari, Simon Kelly, Andrew Cannavan, Dileep Kumar Singh

**Affiliations:** a Soil Microbial Ecology and Environmental Toxicology Laboratory, Department of Zoology, University of Delhi, New Delhi, Delhi, 110007, India; b Food and Environmental Protection Laboratory, International Atomic Energy Agency, Vienna International Centre, PO Box 100, 1400, Vienna, Austria; c Seibersdorf Laboratory, International Atomic Energy Agency, Vienna International Centre, PO Box 100, 1400, Vienna, Austria

**Keywords:** Virgin coconut oil, ICP-MS, Multivariate chemometrics, Regression model

## Abstract

Recently, Virgin coconut oil (VCO) has emerged as one of the most favorable edible oils because of its application in cooking, frying as well as additive used in food, pharmaceuticals, and cosmetic goods. These qualities have established VCO in high consumer demand and there is a great need of establishing a reliable method for the identification of its geographical origin. Through this present study, for the first time, it has been established that Inductively Coupled Plasma-Mass-Spectrometry (ICP-MS) combined with multivariate chemometrics can be used for the identification of the geographical origin of the VCO samples of various provinces. Principal Component Analysis (PCA), and Linear Discriminant Analysis (LDA) were able to differentiate and classify the VCO samples of different geographical origins. Further, calibration models (Principal Component Regression and Partial Least Square Regression) were developed on the calibration dataset of the elemental concentration obtained from the ICP-MS analysis. An external dataset was used to develop the prediction model to predict the geographical origin of an unknown sample. Both PCR and PLS-R models were successfully able to predict the geographical origin with a high R^2^ value (0.999) and low RMSEP value 0.074 and 0.075% v/v of prediction respectively. In conclusion, ICP-MS combined with regression modelling can be used as an excellent tool for the identification of the geographical origin of the VCO samples of various provinces. This whole technique is the most suitable as it has high sensitivity as well as provides easy multi-metal analysis for a single sample of edible oil.

## Highlights

• Virgin coconut oil plays an essential role in our routine life.
• High consumption demand of VCO leads to immense adulteration incidents.
• ICP-MS & chemometrics is an excellent tool for the VCO origin detection.
• PCA, and LDA classified the VCO samples of different geographical origins.
• PCR, and PLS-R models correctly predicted the geographical origin of VCO.

## Graphical abstract

**Figure:**
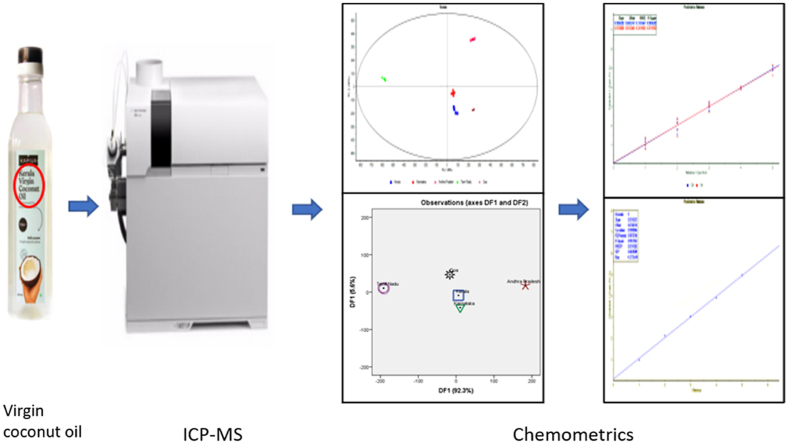

## Abbreviations

ICP-MS: Inductively Coupled Plasma-Mass-Spectrometry
VCO: Virgin Coconut Oil
PCA: Principal Component Analysis
HCA: Hierarchical Cluster Analysis
LDA: Linear Discriminant Analysis
PCR: Principal Component Regression
PLS-R: Partial Least Square Regression
R2: coefficient of determination
RPD: Residual Predictive Deviation
RMSEC: Root Mean Square Error of Calibration
RMSECV: Root Mean Square Error of Cross-Validation
RMSEP: Root Mean Square Error of Prediction

## 1 Introduction

In recent times, Virgin coconut oil (VCO) has arrived as one of the most beneficial edible oil because of its broad range of uses in cooking, frying besides as an additive used in food, pharmacy, and cosmetic goods. It is one of the most advantageous edible oil after olive oil (Marina et al., 2009a). These qualities have established VCO in high consumption demand. The official European Union (EU) classification implemented for defining oil authenticity and quality are “Protected designation of origin” (PDO) and “Protected geographical indication” (PGI) (EEC, 1992). Many studies have been carried out and published on validating the endogenous species as markers of origin of monovarietal oils by many analytical approaches, such as NMR (Mannina et al., 1999; Sacchi et al., 1998), FT-IR (Amit et al., 2020a, 2020b, 2020c) and GC (Benincasa et al., 2003).

In addition to these findings of organic components of oils, heavy metal/element analysis plays a crucial role for edible oil geographical determination and characterization (Zeiner et al., 2005; Benincasa et al., 2007). The existence of metals in edible oils may be because of many aspects: the metals can be assimilated in the edible oil through the soil or production process of the packed edible oils. Hence, it can be stated that elemental allocation in VCO differs corresponding to its origin, and the multi-elemental data subjected to statistical analysis could be used in the geographical identification of VCO samples with different origins.

Atomic absorption spectroscopy (AAS) and inductively coupled plasma mass spectrometry (ICP-MS) are the most frequently applied technology for the determination of multi-metal concentration in different food samples (Zeiner et al., 2005).

Since in the case of edible oils, multi-metals are in very low concentration, therefore it is very difficult to determine multi-metals concentration using AAS. Moreover, the concentration of only a limited number of metals can be determined by AAS. To solve this issue, ICP-MS is the most suitable technique as it has high sensitivity as well as it provides easy multi-metal analysis for a single sample of edible oil (Benincasa et al., 2007).

Since VCO is one of the most valuable edible oils in the market, its geographical origin is indicated on the product by most of the brands and companies to depict its authenticity and quality. This aspect of VCO production becomes very critical for consumers as well as authorities. Keeping this background information in mind, we performed a primary study to determine the geographical origin of different VCO samples using ICP-MS along with multivariate chemometrics.

In our case, VCO samples procured with different origins are subjected to ICP-MS to determine the concentration of twenty trace elements. But merely analysing or comparing multi-metal data did not provide any valuable or conclusive information to determine the geographical origin of different VCO samples. So, the obtained multi-metal concentration data is further subjected to multivariate chemometrics to obtain the valuable information to be used in identifying the geographical distribution of different VCO samples.

Multivariate chemometrics has been exceedingly implemented recently for the analysis of various adulterants in coconut oil (Amit et al., 2020a, 2020b, 2020c). But for geographical identification of different edible oils, there are very few cases where multivariate chemometrics has been applied so far. However, in these studies also, only the differentiation methods (PCA, and LDA) have been used for the geographical identification of edible oils (Benincasa et al., 2007; Aceto et al., 2019). So, there was a gap in the geographical identification studies of edible oils regarding the accuracy and precision of the methodology used till now. But in our case, for the first time, we have utilized regression modelling (PCR, and PLS-R) along with ICP-MS analysis for the geographical identification of VCO samples of different provinces. Moreover, an external set of samples has also been used to predict the geographical origin of an unknown sample. These regression models along with various validation parameters (R^2^, RPD, and RMSE) provided high accuracy and precision in our results. Moreover, there is no study reported in the literature so far where ICP-MS along with multivariate chemometrics has been used to determine the geographical origin of coconut oil. In the present study, Principal Component Analysis (PCA) has been used for obtaining principal components and for the selection of the most informative elements crucial for further analysis. Linear Discriminant Analysis (LDA) has been utilized for classifying and differentiating VCO samples of different origins based on the multi-elemental data. For constructing a suitable regression methodology, Principal Component Regression (PCR) and Partial Least Square Regression (PLS-R) calibration models were used to build the vigorous calibration model. For predicting the geographical origin of the prediction sample set, R^2^, RMSE, and RPD values were examined.

## 2 Materials and methods

### 2.1 Sample procurement

For virgin coconut oil (VCO) samples used in this study, coconut fruit samples were procured from five major coconut-producing states of India i.e., Kerala, Karnataka, Andhra Pradesh, Tamil Nadu, and Goa. with various cultivars as depicted in Table 1. Further virgin coconut oil (VCO) was extracted and stored at 4 °C for further use in the experiment.

**Table 1:** Coconut fruit sample procurement.

Province	No. of samples	Variety
Kerala	05	LCT, WCT, VPM-3, Philippines Ordinary, Kera Sagara
Karnataka	05	WCT, LCT, VPM-3, TPT
Andhra Pradesh	04	WCT, ECT, LCT, Philippines Ordinary
Tamil Nadu	04	VPM-3, ECT, Aligar Nageri, Kera Chandra
Goa	03	LCT, ECT, VPM-3

### 2.2 VCO extraction by cold extraction method

#### 2.2.1 Coconut milk extraction

Testa and coconut water were isolated from the kernel part of the coconut fruit. Freshly obtained kernel part was divided into small pieces and processed through a juicer grinder and coconut milk was obtained. This coconut milk was filtered through a muslin cloth.

#### 2.2.2 VCO extraction

This filtered coconut milk was incubated at 10 °C for 10 h. This incubation led to the solidification of the lipids and the separation of oil globules from the water molecules. Further, the aqueous layer was discarded and the lipid block was incubated at 30 °C until it dissolved completely. This dissolved lipid mixture was centrifuged at 16000 g for 45 min and the oil layer was separated. This obtained oil is pure, without any chemical additives, called virgin coconut oil (VCO) (Seneviratne et al., 2009).

### 2.3 Sample treatment (microwave assisted acid digestion)

Before the ICP-MS analysis, microwave-assisted acid digestion was performed to dissolve the VCO samples using Anton Paar make model (Microwave PRO) oven. Each sample was assiduously mixed and 0.5 g of aliquot was weighed straight into the digestion vessel. The digestion was carried out by adding 5 mL HNO_3_ to each sample. The operating parameters for microwave-assisted acid digestion are depicted in Table 2. After cooling down of all the samples to room temperature, samples were transferred into the volumetric flask, and volume was made up to 20 mL with Milli Q water. A standard calibration curve was recorded with a blank sample spiked with a standard solution having twenty elements (Benincasa et al., 2007).

**Table 2:** Parameters for microwave assisted acid digestion.

Parameter	Microwave Operating Conditions
Sample Volume	1000 uL
Conc. HNO3	8 mL
Internal Temperature Limit (°C)	200
Max. Microwave Power (Watt)	1200
Max. Pressure (bar)	60
Time (min)	30
Volume make-up	40 mL
Filtration of samples	0.2-μm membrane
Number of replicates	3

### 2.4 ICP-MS analysis

The elemental analysis was performed using ICP-MS Spectrometer (Agilent Technologies make Model: 7900) and measurements were recorded in triplicates using a standard calibration curve. The ICP-MS instrumental operating conditions are as follows: The flow of Nebulizer Gas was 1 L/min whereas the auxiliary and plasma gas flow was maintained at 1 L/min and 15 L/min respectively. The reflected and forward power was set at 45 W and 1500 W respectively. Furthermore, the helium gas flow in the reaction was kept at 0.2 mL/min (Table 3).

**Table 3:** ICP-MS instrumental operating conditions for elemental analysis.

Spectrometer	Agilent Technologies make Model: 7900
Nebulizer Gas flow	∼ 1 L/min
Auxiliary Gas flow	∼ 1 L/min
Plasma Gas flow	∼15 L/min
He Gas flow in Reaction Cell	∼ 0.2 mL/min
Reflected Power	∼ 45 W
Forward Power	∼ 1500 W
Analyzer vacuum	∼6 × 10-5

### 2.5 Multivariate chemometric analysis

Statistical software SPSS 20 was used to perform LDA analysis while PCA, HCA, PCR, and PLS-R were performed using Unscrambler 11 software. Principal Component Analysis (PCA) converts many possibly concurrent variables into a few dissimilar factors that are defined as principal components (PCs) and therefore reduces the size of the dataset (Vasconcelos et al., 2015; Amit et al., 2020a). PCA approved the identification of the most crucial variables corresponding with the ICP-MS data of VCO samples of various provinces. The rejection of ineffective variables is essential to get robust and uncomplicated outcomes. Hierarchical cluster analysis (HCA) is an exploratory statistical technique originated to form natural groupings within a data set that would otherwise not be evident. Whereas Linear Discriminant Analysis (LDA) is a statistical method used to obtain a linear amalgamation of forms with the quality to differentiate observation classes (Vasconcelos et al., 2015). Further, the regression models including Principal Component Regression (PCR) and Partial Least Square Regression (PLS-R) are used which are specially devised for the cases having more probably correlated predicting variables than the number of samples. Furthermore, the accuracy and the precision of the predictive model were assessed by measuring the R^2^, RMSE, and the residual predictive deviation (RPD) of the prediction (external) dataset (Amit et al., 2020a). Firstly, PCA was applied to the data collected from the ICP-MS analysis, for obtaining principal components and for selecting the most important elements vital for further analysis. HCA was applied to check whether the VCO samples from the same geographical origin are forming a separate cluster to the samples of different geographical origins. Further, LDA has been used for classifying and differentiating VCO samples of different origins based on the multi-elemental data. Principal Component Regression (PCR) and Partial Least Square Regression (PLS-R) calibration models were constructed to obtain the vigorous calibration model by employing the calibration data set obtained from the ICP-MS analysis. Further, an external data set was used to predict the geographical origin of unknown VCO samples in terms of, R^2^, RMSE, and RPD values.

The efficiency of both the constructed models was analysed and distinguished based on R^2^, RMSEC, and RMSECV values by utilizing the calibration data. In contrast, R^2^ and RMSEP values were employed to check the perdition capability of the constructed model by utilizing an external dataset. The lesser the RMSEP value, the higher the extent of prediction accuracy given by the model and vice-versa for the R^2^ value of prediction. For every model, R^2^, and RMSE were measured for both calibration and prediction datasets, whereas BIAS, SEP, and RPD (must be above 6.5) were measured for the prediction dataset. R^2^ (coefficient of determination) is a statistical unit to determine how close the data are to the fitted regression line. When the regression equation fits the data well, R^2^ will be large (close to one). While RMSE is a parameter to determine how spread out these residuals (data points) are from the regression line. Whereas, Residual Predictive Deviation (RPD) is used to check how well a calibration model can predict. The greater the RPD, the higher the probability of the model to predict the samples outside the calibration set with accuracy and precision. In addition, Standard Error of Prediction (SEP) examines and compares the predictive ability of the regression models. The highest R^2^ (near to one) and least RMSE value make a constructed model most competent. Besides, RPD, SEP, and the BIAS values were estimated, which illustrates the accuracy and precision of the built models.

## 3 Results and discussion

### 3.1 ICP-MS data analysis

The mean elemental composition/Concentration (ppb) of twenty trace elements obtained from the ICP-MS analysis has been depicted in Table 4. Co, Ar, and B were not detected in any of the VCO samples and therefore are not included in the data table for further analysis. Na, Mg, Fe, and P have been detected in high concentrations in almost all the VCO samples of all provinces. The very high concentration of phosphorous in almost all VCO samples of all the states is prominent because of the high use of fertilizers in coconut cultivation. The rest of the elements have varying concentrations in different samples. This ICP-MS elemental data cannot help in discriminating between different provinces for geographical origin identification. Therefore further, this elemental data was fed into different statistical software for multivariate chemometric analysis.

**Table 4:** The mean elemental composition (ppb) of all VCO samples obtained from the ICP-MS spectrometer (Agilent Technologies make Model: 7900).

Province (ppb)	Kerala	Karnataka	Andhra Pradesh	Tamil Nadu	Goa
**Na**	200.42 ± 4.75	298.25 ± 3.61	724.58 ± 1.41	28.38 ± 8.69	286.38 ± 1.53
**Mg**	65.93 ± 3.6	45.50 ± 13.82	146.38 ± 3.02	75.83 ± 3.88	156.14 ± 2.82
**Al**	24.98 ± 7.64	74.89 ± 8.45	69.47 ± 6.16	21 ± 6.67	66.85 ± 6.59
**P**	836.67 ± 2.4	750.1 ± 1.37	674.89 ± 1.22	165.47 ± 7.77	973.28 ± 0.45
**Ca**	32.94 ± 17.04	26.17 ± 15.01	65.76 ± 7.56	30.86 ± 11.18	35.36 ± 12.46
**Cr**	5.92 ± 5.23	14.03 ± 10.39	17.40 ± 19.8	3.54 ± 1.72	7.88 ± 55.91
**Mn**	2.30 ± 2.03	2.36 ± 6.81	4.96 ± 74.83	3.76 ± 1.77	7.24 ± 60.84
**Fe**	109.56 ± 4.34	87.97 ± 6.09	113.77 ± 4	64.87 ± 3.75	129.36 ± 3.4
**Ni**	3.18 ± 7.57	5.12 ± 12.03	8.60 ± 41.72	2.21 ± 7.27	5.01 ± 69.95
**Cu**	1.28 ± 1.45	0.94 ± 7.32	2.90 ± 136.28	0.44 ± 9.83	3.41 ± 34.52
**Zn**	4.54 ± 4.16	21.75 ± 12.15	5.83 ± 64.13	3.09 ± 15.58	3.29 ± 68.78
**Se**	0.18 ± 63.5	0.32 ± 64.62	0.31 ± 39.93	0.28 ± 120.96	0.56 ± 44.25
**Rb**	0.05 ± 3.33	0.16 ± 11.81	0.15 ± 72.89	0.26 ± 4.15	0.37 ± 72.56
**Sr**	0.22 ± 25.3	1.11 ± 28.7	2.46 ± 20.52	0.27 ± 35.88	1.07 ± 23.88
**Mo**	0.14 ± 14.35	0.14 ± 1.47	0.34 ± 33.29	0.05 ± 9.51	0.35 ± 63.82
**Cs**	0.02 ± 24.64	0.03 ± 19.22	0.08 ± 145.04	0.01 ± 23.83	0.27 ± 66.24
**Pb**	0.12 ± 6.98	0.01 ± 8.13	0.20 ± 55.37	0.12 ± 4	0.26 ± 68.61

All values are depicted as mean ± R.S.D.

ppb: parts per billion.

### 3.2 Principal Component Analysis

The ICP-MS data obtained for the five provinces (Kerala, Karnataka, Andhra Pradesh, Tamil Nadu, and Goa) and 17 elements (variables) (Na, Mg, Al, P, Ca, Cr, Mn, Fe, Ni, Cu, Zn, Se, Rb, Sr, Mo, Cs, Pb) was recorded for a total of 21 samples. It is a huge task to reach any substantial conclusion from this dataset without applying any statistics. So, to solve this issue we applied Principal Component Analysis (PCA) to reduce this bulky dataset into principal components. These principal components are those important variables that help us to establish a relationship between the samples and the variables (element concentration) used in the study.

PCA depicts and forms clusters of the data variables into a fewer number of important and unconnected variables called principal components (PCs) having scored for every sample. This obtained score is used to analyse the grouping scheme of various samples in which similar samples are expected to be in the same group. Hence, it is a data reduction methodology depicting the overall scheme of the grouping of data and describes various groups and outliers in the whole dataset (Amit et al., 2020a).

**Fig. 1:**
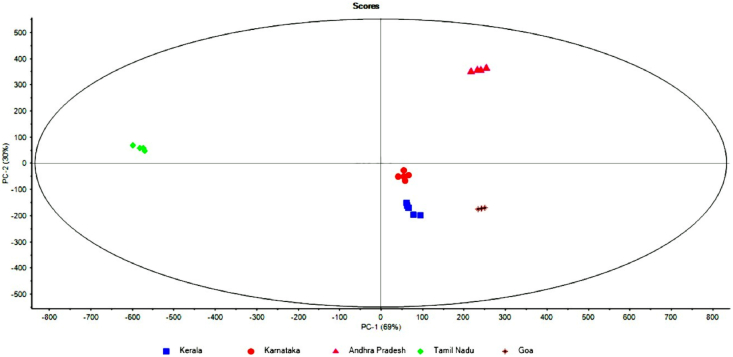
PCA score plot with PC1 and PC2 depicting clear segregation of VCO samples of different provinces based on the ICP-MS dataset of 17 different elements.

From the PCA score plot, a clear pattern of segregation can be observed between VCO samples of various provinces (Fig. 1). This segregation is defined by two principal components (PCs) i.e., principal component 1 (PC1) and principal component 2 (PC2), which explained 69% and 30% of the variance, respectively. Therefore, the first two PCs explained 99% of the total variance, separating all the VCO samples from the various provinces.

### 3.3 Hierarchical Cluster Analysis

For further classification of the ICP-MS data into different clusters, Hierarchical Cluster Analysis (HCA) was performed. HCA classifies the data into different sample groups based on the similarities known as the clusters. HCA puts data samples into one cluster based on the similarities and separates that particular cluster from the samples of another cluster (Richter et al., 2019). In our case, Cluster analysis was applied to determine distance or similarities among the VCO samples and the elements.

**Fig. 2:**
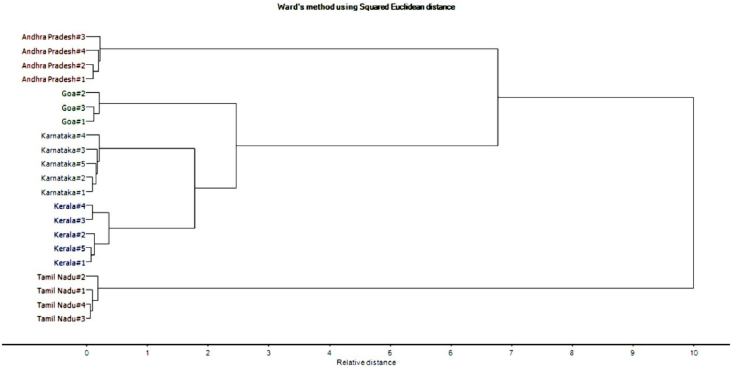
HCA dendrogram depicting the clustering of VCO samples with their respective elemental concentration of different geographical origins (provinces).

Ward's method of linkage with squared Euclidean distance was used as a measure of similarity for the HCA measurement. The HCA output is depicted in the form of a dendrogram (Fig. 2).

It is very evident from the dendrogram that the cluster having samples of Kerala province are nearest to the cluster having samples of Karnataka as the height of linkage branch joining these two is smallest. This linkage branch height represents the distance between two clusters. Further, the common branch of the above clusters is linked closest to the cluster having VCO samples of Goa province. In accordance, the common branch of all three clusters is joined to the cluster with VCO samples of Andhra Pradesh province. And at last, the common branch arising from all these four clusters is distantly linked to the cluster with samples of Tamil Nadu province. Therefore, from this HCA analysis, it can be seen that the VCO sample of all the provinces are making different clusters. Moreover, the HCA cluster of Kerala province is closed to the Karnataka samples and in turn closer to the Goa samples. While Andhra Pradesh and Tamil Nadu samples are making clusters, which are distantly situated. This HCA dendrogram result is in accordance with the PCA score plot analysis.

### 3.4 Linear Discriminant Analysis (LDA)

LDA was applied for further classification and discriminative analysis of VCO samples based on their geographical origin. PCs imparting to the variation in the dataset were exposed to discriminant analysis using “IBM SPSS Statistics 20” to determine the possibility of a sample of a formerly determined cluster. LDA is a statistical method used to obtain a linear amalgamation of forms with the quality to differentiate observation classes (Vasconcelos et al., 2015). LDA firstly generates a classification model by employing a calibration (training) dataset, and later this model is used for the prediction of unknown samples using a separate validation dataset. In the majority of cases, with an incomplete number of samples, the cross-validation method is implemented, which lacks to develop separate validation dataset. In the cross-validation approach, the calibration dataset is used as a validation dataset for the validation of model efficiency. LDA methodology uses linear Euclidean distance to reduce within-class variance and increase the gap between classes. For the selection of the optimal number of discriminant factors in the LDA model, the Leave one out cross-validation (LOOCV) method is employed within the estimated classes.

**Fig. 3:**
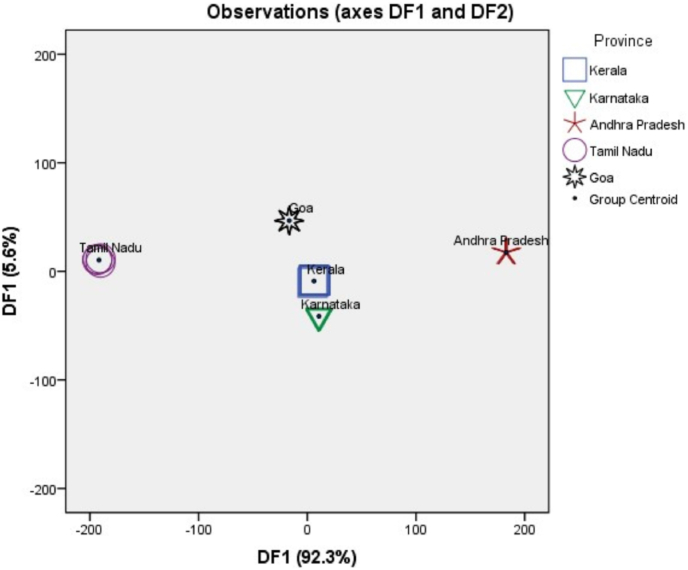
All groups scatter plot as deduced by discriminant analysis using discriminant function 1 and 2 for the differentiation of VCO samples of different provinces based on their geographical origin.

In our case, Both the discriminant functions with Eigenvalue >1 and p values < 0.001 are significant and demonstrate 92.3% and 5.6% variance of the VCO samples of various provinces respectively. All groups scatter plot, as deduced by LDA using discriminant function 1 and 2 for VCO samples also explains the total variance (Fig. 3). The group centroid represented in the plot depicts that function 1 and function 2 are differentiating between the VCO samples of various provinces based on their elemental composition which depicts their geographical origin. The confusion matrix resulting from the LDA classified 100% of the initial groups as well as classified correctly when cross-validated as shown in Table 5. In cross-validation, each case is classified by the functions derived from all cases except that case.

**Table 5:** Confusion matrix for the classification of VCO samples of different provinces based on their geographical origin.

		Province	Predicted Group Membership					Total
			Kerala	Karnataka	Andhra Pradesh	Tamil Nadu	Goa	
Original	Count	Kerala	5	0	0	0	0	5
		Karnataka	0	5	0	0	0	5
		Andhra Pradesh	0	0	4	0	0	4
		Tamil Nadu	0	0	0	4	0	4
		Goa	0	0	0	0	3	3
	%	Kerala	100	0	0	0	0	100
		Karnataka	0	100	0	0	0	100
		Andhra Pradesh	0	0	100	0	0	100
		Tamil Nadu	0	0	0	100	0	100
		Goa	0	0	0	0	100	100
Cross-validated	Count	Kerala	5	0	0	0	0	5
		Karnataka	0	5	0	0	0	5
		Andhra Pradesh	0	0	4	0	0	4
		Tamil Nadu	0	0	0	4	0	4
		Goa	0	0	0	0	3	3
	%	Kerala	100	0	0	0	0	100
		Karnataka	0	100	0	0	0	100
		Andhra Pradesh	0	0	100	0	0	100
		Tamil Nadu	0	0	0	100	0	100
		Goa	0	0	0	0	100	100

From the outcome, it has been observed that the VCO samples belonging to a particular class (province) are well classified and differentiated from the samples of another class. These LDA results are in accordance with the pattern observed in both PCA and HCA analysis. Further to strengthen our results obtained from the above approaches (PCA, HCA, and LDA), PCR and PLS-R regression models were developed from the calibration dataset obtained from the ICP-MS analysis. And the constructed models were further validated by predicting the models using the external dataset which was not used in the calibration model.

### 3.5 Prediction of the geographical origin of VCO samples by regression models based on elemental data

For the prediction of the geographical origin of VCO samples of different provinces, PCR and PLS-R regression models were constructed based on elemental data obtained from the ICP-MS analysis. In most of the linear regression and prediction cases, the independent variables may be highly collinear known as multicollinearity. PCR resolves the co-linearity problem with lesser factors. Although, PLS-R may even resolve the problem with fewer factors than PCR. Simulations have depicted that PLS-R provides its least root mean square error (RMSE) with fewer factors than PCR (Yeniay and Goktas, 2002). These regression methods bank on two steps which are called as calibration and prediction. For the calibration step, a regression model was constructed to set up a relation between the ICP-MS elemental concentration (predictor variables) and the different provinces or geographical origin (response variable), using the calibration set of samples. And in the prediction step, the constructed model was utilized to measure the geographical origin of an external set of samples that were not used in model development. The optimal number of factors was established by employing the Leave-One-Out method for cross-validation. It is calculated from the plot between the number of factors and the RMSECV which gives an optimal number of factors for both the models (Rohman et al., 2017). The optimum number of factors plays major role in reducing the RMSECV value. The competence of the developed models for the prediction of the geographical origin for an external set of samples was examined by the RMSEP value. The relation between the number of factors and the RMSEC is inversely proportional to each other. A model constructed with a greater number of factors would result in overfitting, resulting in the very low RMSEC but very high RMSEP values. The prediction capability of the constructed model is checked using the R^2^ and the RMSEP (root mean square error of prediction). The lesser the RMSEP value, the higher the ability to predict accurate model and vice-versa for the R^2^ value of prediction (Rohman et al., 2017). For both the models, R^2^ and RMSE were estimated for both calibration and prediction datasets, whereas BIAS, SEP, and RPD (must be above 6.5) were estimated for the prediction dataset. Table 6 shows the number of factors corresponding to the least RMSE and RMSECV values of both models.

**Table 6:** PCR and PLS-R models for the prediction of the geographical origin of the VCO samples by using the elemental concentrations obtained from the ICP-MS analysis.

				aR^2^			RMSE				
Model	Factor		Calibration	Validation	Prediction	bRMSE	cRMSECV	dRMSEP	gRPD	BIAS	hSEP
ePCR	05		0.983	0.971	0.997	0.176	0.241	0.074	7.83	-4.27	0.082
fPLS-R		05	0.985	0.973	0.997	0.167	0.240	0.075	7.74	0.007	0.083

a R2: Coefficient of determination.

b RMSEC: Root mean square error of calibration.

c RMSECV; Root mean square error of cross-validation.

d RMSEP; Root mean square error of prediction.

e PCR: Principal component regression.

f PLS-R: Partial least squares regression.

g RPD: Residual Predictive Deviation.

h SEP: Standard Error of Prediction.

**Fig. 4:**
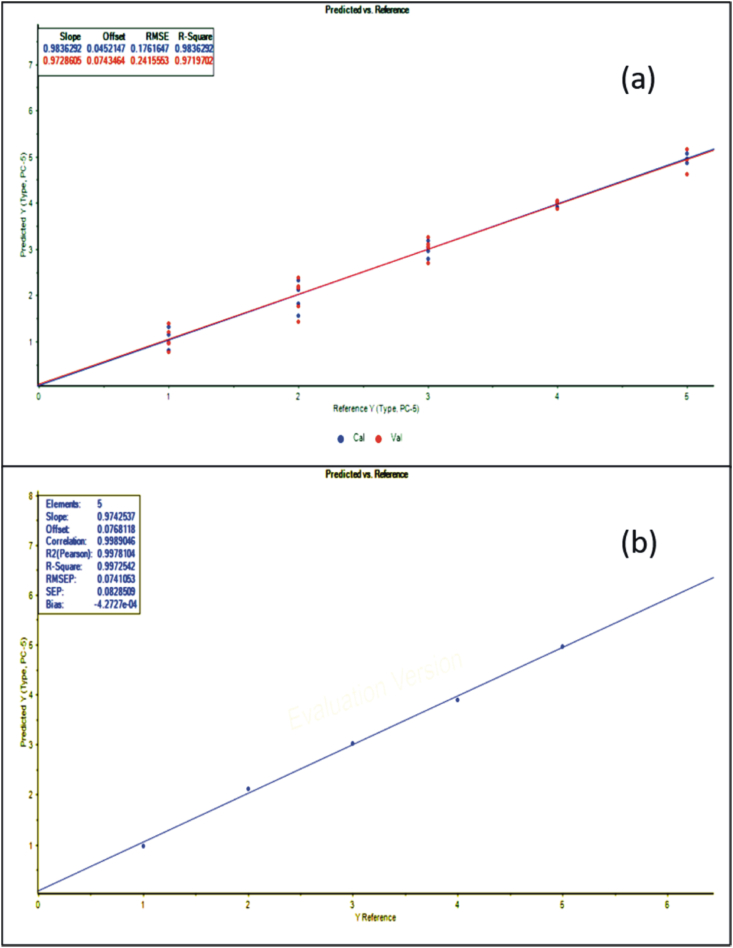
(a) Principal Component Regression (PCR) calibration model of calibration set of VCO samples for a relationship between actual (Reference Y) versus predicted (Predicted Y) geographical origin using the elemental concentrations obtained from the ICP-MS analysis (Here 1- Kerala, 2 - Karnataka, 3 – Andhra Pradesh, 4 – Tamil Nadu, 5 – Goa) **(b)** Principal Component Regression (PCR) prediction model of an external set of VCO samples.

**Fig. 5:**
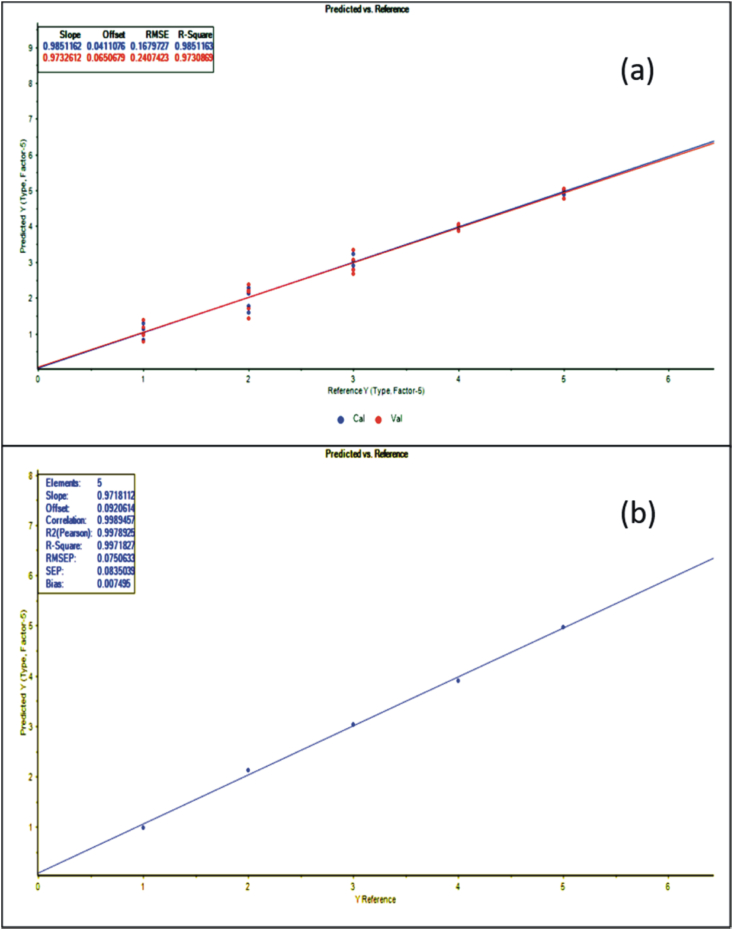
(a) Partial least squares regression (PLS-R) calibration model of calibration set of VCO samples for a relationship between actual (Reference Y) versus predicted (Predicted Y) geographical origin using the elemental concentrations obtained from the ICP-MS analysis (Here 1- Kerala, 2 - Karnataka, 3 – Andhra Pradesh, 4 – Tamil Nadu, 5 – Goa) **(b)** Partial least squares regression (PLS-R) prediction model of an external set of VCO samples.

Fig. 4 and Fig. 5 depict the graphs of the measured geographical origin versus the predicted geographical origin from the ICP-MS data, which manifests the accuracy and significance of the developed models. Table 6 also demonstrates the different quality aspects (accuracy and precision) of the two calibration models in the forms of R^2^, RPD, and RMSE values. The relationship between measured and predicted geographical origin of elemental concentration based on ICP-MS analysis for PCR and PLS-R shows R^2^ value 0.983 and 0.985 respectively for calibration and 0.997 when the external set was used for prediction. And RMSEC value are 0.176 and 0.167% v/v for calibration and 0.074 and 0.075% v/v for prediction (Table 6). This can also be explained from the above findings that both the PCR and PLS-R models are successfully able to predict the geographical origin of the VCO samples by using the elemental concentrations obtained from the ICP-MS analysis.

## 4 Conclusion

Through this present study, for the first time, it has been established that ICP-MS elemental data combined with multivariate chemometric tools can be used for the identification of the geographical origin of the VCO samples of various provinces. This whole technique is the most suitable as it has high sensitivity as well as provides easy multi-metal analysis for a single sample of edible oil. PCA, HCA and LDA were able to differentiate and classify the VCO samples of different geographical origins. Further, calibration models (PLS-R and PCR) were developed on the calibration dataset of the elemental concentration obtained from the ICP-MS analysis. An external dataset was used to develop the prediction model to predict the geographical origin of an unknown sample. Both PCR and PLS-R models were successfully able to predict the geographical origin with a high R^2^ value (0.999) and low RMSEP value 0.074 and 0.075% v/v of prediction respectively. The performance of the calibration models was analysed by using an external set of data which gave a low relative error and high (above 6.5) residual predictive deviation, resulting in high accuracy and precision. In conclusion, ICP-MS combined with regression modelling can be used as an excellent tool for the identification of the geographical origin of the VCO samples.

## CRediT authorship contribution statement

**Amit:** Writing – original draft. **Rahul Jamwal:** Writing – review & editing. **Shivani Kumari:** Investigation, Data curation. **Simon Kelly:** Formal analysis. **Andrew Cannavan:** Formal analysis. **Dileep Kumar Singh:** Supervision, Project administration.

## Declaration of competing interest

The authors declare that they have no known competing financial interests or personal relationships that could have appeared to influence the work reported in this paper.

## References

[bib1] Aceto M., Calà E., Musso D., Regalli N., Oddone M. (2019). A preliminary study on the authentication and traceability of extra virgin olive oil made from Taggiasca olives by means of trace and ultra-trace elements distribution. Food Chem..

[bib2] Amit, Jamwal R., Kumari S., Dhaulaniya A.S., Balan B., Singh D.K. (2020). Application of ATR-FTIR spectroscopy along with regression modelling for the detection of adulteration of virgin coconut oil with paraffin oil. LWT (Lebensm.-Wiss. & Technol.).

[bib3] Amit, Jamwal R., Kumari S., Dhaulaniya A.S., Balan B., Kelly S., Cannavan A., Singh D.K. (2020). Utilizing ATR-FTIR spectroscopy combined with multivariate chemometric modelling for the swift detection of mustard oil adulteration in virgin coconut oil. Vib. Spectrosc..

[bib4] Amit, Jamwal R., Kumari S., Kelly S., Cannavan A., Singh D.K. (2020). Rapid detection of pure coconut oil adulteration with fried coconut oil using ATR-FTIR spectroscopy coupled with multivariate regression modelling. LWT (Lebensm.-Wiss. & Technol.).

[bib5] Benincasa C., De Nino A., Lombardo N., Perri E., Sindona G., Tagarelli A. (2003). Assay of aroma active components of virgin olive oils from southern Italian regions by SPME-GC/ion trap mass spectrometry. J. Agric. Food Chem..

[bib6] Benincasa C., Lewis J., Perri E., Sindona G., Tagarelli A. (2007). Determination of trace element in Italian virgin olive oils and their characterization according to geographical origin by statistical analysis. Anal. Chim. Acta.

[bib7] Mannina L., Patumi M., Fiordiponti P., Emanuele M.C., Segre A.L. (1999). Olive and hazelnut oils: a study by high-field 1H NMR and gas chromatography. Ital. J. Food Sci..

[bib8] Marina A.M., Che Man Y.B., Nazimah S.A.H., Amin I. (2009). Chemical properties of virgin coconut oil. J. Am. Oil Chem. Soc..

[bib9] Regulation C. (1992). Council Regulation (EEC) No. 2081/92 of 14 July 1992 on the protection of geographical indications and designations of origin for agricultural products and foodstuffs. Off. J. Eur. Union.

[bib10] Richter B., Gurk S., Wagner D., Bockmayr M., Fischer M. (2019). Food authentication: multi-elemental analysis of white asparagus for provenance discrimination. Food Chem..

[bib11] Rohman A., Che Man Y.B., Ismail A., Hashim P. (2017). FTIR spectroscopy coupled with chemometrics of multivariate calibration and discriminant analysis for authentication of extra virgin olive oil. Int. J. Food Prop..

[bib12] Sacchi R., Mannina L., Fiordiponti P., Barone P., Paolillo L., Patumi M., Segre A. (1998). Characterization of Italian extra virgin olive oils using 1H-NMR spectroscopy. J. Agric. Food Chem..

[bib13] Seneviratne K.N., HapuarachchI C.D., Ekanayake S. (2009). Comparison of the phenolic-dependent antioxidant properties of coconut oil extracted under cold and hot conditions. Food Chem..

[bib14] Vasconcelos M., Coelho L., Barros A., de Almeida J.M.M.M. (2015). Study of adulteration of extra virgin olive oil with peanut oil using FTIR spectroscopy and chemometrics. Cogent Food Agric..

[bib15] Yeniay O., Goktas A. (2002). A comparison of partial least squares regression with other prediction methods. Hacettepe J. Math. Stat..

[bib16] Zeiner M., Steffan I., Cindric I.J. (2005). Determination of trace elements in olive oil by ICP-AES and ETA-AAS: a pilot study on the geographical characterization. Microchem. J..

